# The relative age effect among female basketball players in the Israeli Premier League

**DOI:** 10.3389/fspor.2025.1644415

**Published:** 2025-08-29

**Authors:** Simcha Avugos, Michal Malul

**Affiliations:** Levinsky-Wingate Academic Center—Wingate Campus, Netanya, Israel

**Keywords:** relative age effect, female athletes, talent development, elite sports, selection bias

## Abstract

In sports, the term *relative age effect* refers to the asymmetrical distribution of athletes based on their birth dates relative to an arbitrary cut-off date. Some studies indicate that athletes who were born shortly after this cut-off tend to have higher representation in elite sports leagues compared to those who were born later in the year. Yet the literature presents inconsistencies in empirical support for this effect. The aim of this study, therefore, was to examine the relative age effect in female basketball players from the Israeli Premier League, while distinguishing between domestic and foreign players (*n* = 215, M_age_ = 24.08 years, SD = 5.17; and *n* = 120, M_age_ = 30.33 years, SD = 3.68, respectively), and examining two alternative cut-off dates (January 1 and September 1). Data were collected over six seasons, 2018–2024. Chi-square values and odds ratios were calculated to examine the distribution of birth quarters compared to uniform distribution in general, and to Israeli and U.S. live birth data. The findings reveal that the relative age effect was insignificant among the players, regardless of their nationality. While a higher number of players were found to have been born in the second quarter of the year, this difference was statistically insignificant, regardless of whether a uniform distribution of births or normative population values were applied. As such, the findings of the current study do not support the existence of selection bias among coaches based on the birth dates of female professional basketball players in Israel.

## 1 Introduction

Basketball is among the most widely played and followed sports globally, with an estimated 610 million participants across various levels (1). Its popularity is especially strong in the U.S., where the National Basketball Association (NBA) holds significant cultural and commercial influence (2). International competitions such as the FIBA World Cup and the Olympic Games further highlight the sport's global reach and appeal (3).

Recent years have seen a growing emphasis on gender equality in sport. The Paris 2024 Olympic Games, for the first time, featured equal representation of male and female athletes (4). This milestone aligns with broader efforts to promote women's participation in competitive sports (5). In basketball, female participation has steadily increased, supported by grassroots initiatives, national programs, and international events such as the FIBA Women's Basketball World Cup (6), which likely inspire young female talent to participate in this sport. According to FIBA (7), over 500,000 female basketball players are registered with the federation, reflecting the sport's growing inclusivity. Despite persistent challenges such as unequal funding and limited media exposure (8, 9), the outlook for women's basketball continues to improve.

While the study of female basketball players has expanded in recent years (6), particularly as their participation and visibility has increased, the question of how birth timing affects children's development has been explored for nearly a century (e.g., 10–13). Early work by Huntington (14) suggested that factors such as season of birth, birth order, and parental background could influence a child's long-term potential. Building on this foundation, more recent studies have investigated how school entry cut-off dates, which group children born within the same 12-month period, can lead to developmental and academic disparities. Typically, older children within a cohort tend to outperform their younger peers, a phenomenon known as the Relative Age Effect [RAE; (15, 16)]. These early advantages may have long-lasting effects, influencing educational paths, levels of engagement, and even future career choices (17, 18).

In sport, RAE refers to the developmental advantages of being born earlier in the selection year, which can lead to differences in physical, cognitive, and emotional maturity among youth athletes (19). These differences are especially pronounced during adolescence, a period marked by significant biological and psychological changes. Coaches and selectors may unintentionally favor older athletes within an age group, thus reinforcing early advantages (20).

RAE has been widely documented across many sports, particularly those requiring contact and strength (21, 22); see review by (23), including basketball (e.g., 24, 25), soccer (e.g., 26, 27), handball (e.g., 28, 29), baseball (e.g., 30, 31), American football (e.g., 32), and hockey (e.g., 33, 34). It is also present in non-contact and individual sports, such as tennis (e.g., 35), swimming (e.g., 36), and athletics (e.g., 37). In basketball specifically, RAE has been linked to youth selection processes and long-term athlete development pathways (24), raising questions about equity and talent identification practices.

The selection imbalance seen in youth sports can be attributed to several developmental and environmental factors. Older athletes within an age cohort often possess superior motor skills (e.g., balance, coordination, speed, and strength), more advanced physical development (e.g., height, muscle mass) and greater aerobic fitness (38). These early advantages can lead to selection for elite teams, where access to better coaching and training further amplifies their development (39, 40). Conversely, relatively younger athletes within the same birth year may be overlooked, limiting their opportunities for progression.

At the theoretical level, Hancock et al. (41) identified three key social influences on RAE: parents (Matthew effect—where early advantages lead to further gains), coaches (Pygmalion effect—where expectations from authority figures shape outcomes), and the athletes themselves (Galatea effect—where self-belief enhances achievement). These mechanisms highlight the combined influence of external expectations (from others) and internal belief (in oneself) in shaping athletic development. Specifically, when parents and coaches expect older athletes to perform better than their younger peers, the athletes internalize these expectations, which in turn elevate their self-belief and performance. Similarly, Wattie et al. (42) proposed a conceptual model based on Newell's (43) three constraints framework, suggesting that RAE is shaped by the interaction of individual characteristics, task demands, and environmental context.

Despite these insights, the empirical evidence for RAE remains mixed. Several studies report no clear RAE patterns (e.g., 44–47), and some even suggest a counter-trend, where athletes born later in the selection year outperform their older peers over time—a phenomenon known as the “reversal of the RAE advantage” (48, 49). Younger athletes may develop compensatory traits such as adaptability, persistence, and refined technical skills, which support their long-term success. In Israel, studies have shown little to no evidence of RAE among female athletes. Research by Lidor et al. (50–52) across multiple sports found RAE mainly among male participants, while female athletes appeared unaffected. These findings may reflect more inclusive selection practices that prioritize long-term potential over immediate physical advantages.

Given the limited research on female basketball players (6) and the inconclusive findings regarding RAE, the current study aims to examine the presence of the effect among female athletes in the Israeli Basketball Premier League. The study also considers differences between domestic and foreign players and test two distinct cut-off dates for age grouping. In doing so, it contributes to the broader understanding of RAE in women's sports and supports discussions on equity and access in talent development. The finding may offer valuable insights for coaches, sport organizations, and policymakers seeking to create more equitable and inclusive athletic environments.

## 2 Methods

### 2.1 Data collection

To conduct this study, data were retrieved from the official website of the Israeli Women's Premier Basketball League (https://wbi.co.il). A total of 15 teams had participated in at least one season during the 2018–2024 period. Microsoft Excel 2016 (Microsoft Corp., Redmond, WA, USA) was used to document and code the collected data. After creating a database of the relevant teams, players, and seasons, we added background data for each player, including position, height, nationality, and date of birth. To enhance reliability, all data were cross-referenced with publicly available information on other online platforms, and two independent coders were involved throughout the data collection and coding process.

Tracking and compiling the player list over the seasons proved somewhat challenging, as some foreign players had obtained Israeli nationality while still playing on the league, others had transferred to a different team, or had dropped out, and new players were continually recruited. For the purpose of this research, players were documented as domestic (Israeli) or foreign (non-Israeli) based on their citizenship status when they first began playing on the Israeli league. Moreover, records of athletes who had played for more than one team across the six seasons were removed (*n* = 192), and only their first appearance was retained to avoid duplication. This approach ensured that each player was represented only once in the dataset. The final sample consisted of 335 female players.

### 2.2 Data analysis

Data analysis was conducted using SPSS v. 29.0 software (IBM, Inc., NY). In addition to descriptive statistics (age, height, position, and nationality), the chi-square (*χ*^2^) goodness-of-fit test was employed, to determine whether the distribution of the players' birth dates was uniform, evaluated by quarters (the null hypothesis). Cut-off dates were set as January 1 (the beginning of the calendric year) and as September 1 (the start of the school year in many countries; (26, 53, 54). September is a common cut-off date, which is typically where RAE originates (55). To assess differences between birth quarter distributions for the significant *χ*^2^ outputs, Cramer's V value was also calculated, as suggested by others (e.g., 52, 53). The thresholds for interpreting the Cramer's V effect size were as follows: weak ≥.06, moderate ≥.17, and strong ≥.29 (56). Additionally, odds ratios (OR) and 95% confidence intervals (CI) were calculated to compare birth quartiles for the observed and expected distributions. Significance levels were set at *p* ≤ .05 for all tests.

School entry cut-off dates vary across countries and sometimes even within regions of the same country. In Israel, a 1959 law established September 1 as the start of the school year, requiring children born between January 1 and December 31 of the same calendar year to begin first grade, entering school between ages 5 and 6. In the U.S., data from the Education Commission of the States (https://www.ecs.org) shows that most states have consistently adhered to a September cut-off, which has remained unchanged for decades. This consistency reinforces our selected cut-off date and generates further confidence in the reported findings.

Finally, to assess whether the observed birth date distribution reflects true selection effects rather than natural demographic trends, we compared the distribution of players' birth dates by quarters to national live birth data from Israel and the U.S. This comparison helps ensure that any overrepresentation is not simply due to more children being born in a particular season. For Israeli players in the sample, birth data were obtained from the Central Bureau of Statistics (CBS; https://www.cbs.gov.il/EN/Pages/default.aspx). For American players, data were sourced from reports published by the Centers for Disease Control and Prevention (CDC; https://www.cdc.gov.us). Players with other citizenships were excluded from this analysis due small sample size (*n* = 21) and the wide range of countries represented, which made comparable birth distribution data impractical to obtain.

## 3 Results

### 3.1 Descriptive statistics—background data

Table 1 presents the positions of the female players on the field, categorized by nationality (Israeli/foreign). More than one-third of all players (*n* = 120, 35.8%) were foreign players, with about 83% of them (*n* = 99) holding U.S. citizenship. The foreign players exhibited a significant height advantage, ranging from 1.65–2.06 meters (M = 183.21 cm, SD = 8.22), compared to Israeli players, whose heights ranged from 1.60–1.95 meters (M = 174.23 cm, SD = 7.59; *p* < .05). A height of ≥1.90 meters was seen in 27 of the 120 non-Israeli players, compared to only 10 in the 215 Israeli players.

**Table 1:** Background characteristics by nationality of the female players in the Israeli Premier Basketball League, 2018–2024.

Nationality	N	Average Age (SD)	Average Height (SD)	Position in the field							
				Guard	Forward	Small Forward	Guard-Small Forward	Guard-Forward	Power- Forward	Forward-Center	Center
Israeli	215	24.08 (5.17)	174.23 (7.59)	141	47	6	2	7	2	2	8
Other	120	30.33 (3.68)	183.21 (8.22)	44	38	2	0	7	1	10	18
All players	335	26.32 (5.56)	177.47 (8.92)	185	85	8	2	14	3	12	26

Moreover, on average, the foreign players were also significantly older than their Israeli counterparts (M = 30.33, SD = 3.68; and M = 24.08, SD = 5.17, respectively; *p* < .05). The youngest foreign and Israeli players were 20.44 years and 16.12 years, respectively, while the oldest foreign and Israeli players were 40.59 years and 39.74 years, respectively. Regardless of nationality, the following age distribution for all players who were included in the sample was seen, by year of birth: 1984–1989, *n* = 25; 1990–1999, *n* = 168; and 2000–2008, *n* = 142.

### 3.2 Empirical RAE analysis

In the first phase of the analysis, we assumed an equal distribution of births across the months of the year. The 12 months were divided into four birth quarters, starting from *January* 1. Based on the exact number of days in each month, the distribution of birth dates per quartile was as follows: Q1 (January–March), 24.7%; Q2 (April–June), 24.9%; Q3 (July–September), 25.2%; and Q4 (October–December), 25.2%. This distribution served as the expected distribution for the *χ*^2^ tests.

Table 2 presents the number and percentage of players by quartile, together with the results of the *χ*^2^ test. As shown in Table 2A, a peak birth rate was observed in Q2, for all three categories: 1. the entire sample, regardless of nationality (28.06%); 2. the Israeli players (28.37%); and 3. the foreign players (27.50%). Moreover, the highest number of births was seen in the month of August, while the lowest number was recorded in November. However, while this pattern was documented in two of the three categories: the overall sample (*n* = 35 and *n* = 20, respectively), and the Israeli players (*n* = 24 and *n* = 8, respectively), it was not seen in the foreign players.

**Table 2:** Distribution of players' birth month by nationality and statistical analysis. Cut-off dates: January 1 and September 1.

Quartile distribution	(A). Quartile and number and percentage of players (Cut-off date: January 1)								
	Jan–Mar	Apr–Jun	Jul–Sep	Oct–Dec	Total	*χ*^2^ (df = 3)	*p*	Cramer's V	Q1 vs. Q4: OR (95% CI)
	24.7%	24.9%	25.2%	25.2%					
All players	80	94	89	72	335	3.48	.324	0.07	1.14
(%)	(23.88)	(28.06)	(26.57)	(21.49)					(0.732; 1.763)
Expected	82.60	83.52	84.44	84.44					
Israeli players	52	61	61	41	215	5.11	.164	0.11	1.30
(%)	(24.19)	(28.37)	(28.37)	(19.07)					(0.742; 2.264)
Expected	53.01	53.60	54.19	54.19					
Other players	28	33	28	31	120	0.59	.899	0.05	0.92
(%)	(23.33)	(27.50)	(23.33)	(25.83)					(0.450; 1.896)
Expected	29.59	29.92	30.25	30.25					
Quartile distribution	(B). Quartile and number and percentage of players (Cut-off date: September 1)								
	Sep–Nov	Dec–Feb	Mar–May	Jun–Aug	Total	χ^2^ (df = 3)	*p*	Cramer's V	Q1 vs. Q4: OR (95% CI)
	24.9%	24.7%	25.2%	25.2%					
All players	74	80	91	90	335	2.04	.563	0.06	0.83
(%)	(22.09)	(23.88)	(27.16)	(26.87)					(0.540; 1.280)
Expected	83.52	82.60	84.44	84.44					
Israeli players	44	51	62	58	215	3.19	.363	0.09	0.77
(%)	(20.47)	(23.72)	(28.84)	(26.98)					(0.445; 1.322)
Expected	53.60	53.01	54.19	54.19					
Other players	30	29	29	32	120	0.16	.983	0.03	0.95
(%)	(25.00)	(24.17)	(24.17)	(26.67)					(0.466; 1.927)
Expected	29.92	29.59	30.25	30.25					

OR, odds ratio; CI, confidence interval.

* *p* ≤ .05.

No significant differences were seen in the players' actual birth quarter distribution compared to the calculated uniform distribution [*n* = 335, *χ*^2^_(df_ _=_ _3)_ = 3.48, *p* = .324, V = 0.07]. Similarly, no significant deviations from the expected number of births in each quartile were observed for the Israeli players [*n* = 215, *χ*^2^_(df_ _=_ _3)_ = 5.11, *p* = .164, V = 0.11] or for the foreign ones [*n* = 120, *χ*^2^_(df = 3)_ = 0.59, *p* = .899, V = 0.05]. Additionally, no significant ORs were found between birth-quarter distributions in any of the analyses.

Next, we applied an alternative cut-off date for the quarters, starting from *September* 1, as shown in Table 2B. Again, no significant differences were seen in the players' birth quarter distribution compared to the uniform distribution across the three categories: total sample [*n* = 335, *χ*^2^_(df_ _=_ _3)_ = 2.04, *p* = .563, V = 0.06], Israeli players [*n* = 215, *χ*^2^_(df_ _=_ _3)_ = 3.19, *p* = .363, V = 0.09], and foreign players [*n* = 120, *χ*^2^_(df_ _=_ _3)_= 0.16, *p* = . 983, V = 0.03]. Moreover, no significant ORs were found between birth quarter distributions in any of the analyses (OR = 0.83, 0.77, and 0.95, respectively).

### 3.3 RAE analysis vs. normative population values

In this phase of the study, we compared the distribution of birth dates (by quarters) of the Israeli players (*n* = 215) to those of the general Israeli public, as reported by the CBS, for 1984–2008. A similar comparison was also conducted for the foreign players with American citizenship (*n* = 99), for 1984–2001.

Table 3 presents the number and percentage of athletes by quartiles, together with the results of the chi-square test. We started with January 1 as the cut-off date. As shown in Table 3A, no significant differences were found in the distribution of birth quarters for players from the sample compared to that of the general population, for both the Israeli players [*χ*^2^_(df_ _=_ _3)_ _=_ 6.68, *p* = .083, V = 0.12] and the foreign players [*χ*^2^_(df_ _=_ _3)_ = 0.18, *p* = .981, V = 0.03]. Furthermore, no significant ORs were identified between birth quarter distributions in any of the analyses. These results suggest that the distribution of birth dates among the players in the sample, across the different quarters, is in line with that of the general respective populations.

**Table 3:** Distribution of players' birth month compared to the Israeli and U.S. general population and statistical analysis. Cut-off dates: January 1 and September 1.

Q distribution [Israel birth data]	(A) Quartile and number and percentage of players (Cut-off date: January 1)								
	Jan–Mar	Apr–Jun	Jul–Sep	Oct–Dec	Total	χ^2^ (df = 3)	*p*	Cramer's V	Q1 vs. Q4: OR (95% CI)
	24.1%	23.2%	26.6%	26.0%					
Israeli players	52	61	61	41	215	6.68	.083	0.12	1.37
(%)	(24.19)	(28.37)	(28.37)	(19.07)					(0.785; 2.391)
Expected	51.82	49.98	57.25	55.96					
Q distribution [U.S. birth data]	Jan–Mar	Apr–Jun	Jul–Sep	Oct–Dec	Total	χ^2^ (df = 3)	*p*	Cramer's V	Q1 vs. Q4: OR (95% CI)
	24.1%	24.8%	26.4%	24.8%					
American players	23	26	25	25	99	0.18	.981	0.03	0.95
(%)	(23.23)	(26.26)	(25.25)	(25.25)					(0.426; 2.107)
Expected	23.81	24.51	26.15	24.53					
Q distribution [Israel birth data]	(B) Quartile and number and percentage of players (Cut-off date: September 1)								
	Sep–Nov	Dec–Feb	Mar–May	Jun–Aug	Total	χ^2^ (df = 3)	*p*	Cramer's V	Q1 vs. Q4: OR (95% CI)
	26.0%	24.9%	23.4%	25.7%					
Israeli players	44	51	62	58	215	5.51	.138	0.11	0.75
(%)	(20.47)	(23.72)	(28.84)	(26.98)					(0.438; 1.289)
Expected	55.89	53.50	50.28	55.34					
Q distribution [U.S. birth data]	Sep–Nov	Dec–Feb	Mar–May	Jun–Aug	Total	χ^2^ (df = 3)	*p*	Cramer's V	Q1 vs. Q4: OR (95% CI)
	25.2%	24.0%	24.8%	26.0%					
American players	26	22	24	27	99	0.24	.970	0.04	0.99
(%)	(26.26)	(22.22)	(24.24)	(27.27)					(0.460; 2.149)
Expected	24.94	23.72	24.57	25.76					

OR, odds ratio; CI, confidence interval.

* *p* ≤ .05.

Finally, we reconducted the chi-square test, this time using the September 1 alternative cut-off date. As shown in Table 3B, no significant differences were found in the distribution of birth quarters compared to the general population, for Israeli players [*χ*^2^_(df_ _=_ _3)_ = 5.51, *p* = .138, V = 0.11] or for foreign ones [*χ*^2^_(df_ _=_ _3)_ = 0.24, *p* = .970, V = 0.04]. Furthermore, no significant ORs were identified between birth quarter distributions in any of the analyses (OR = 0.75 and 0.99, respectively).

## 4 Discussion

### 4.1 General discussion

By analyzing six seasons of data and distinguishing between Israeli and foreign players, this study assessed whether birthdates among elite women's basketball players are evenly distributed or influenced by selection biases related to age-based cut-off dates. Using national birth statistics from Israel and the U.S. as normative baselines, the findings revealed no significant RAE in this cohort—challenging widely held assumptions about age-related advantages in youth and professional sport. Notably, these results align with previous research on Israeli athletes. For example, Lidor et al. (52) found no evidence of RAE among Israeli female athletes across ten sports, including both team sports (basketball, soccer, handball, volleyball, and water polo), and individual sports (gymnastics, judo, swimming, tennis, and track and field). In contrast, RAE was observed among Israeli male athletes in four sports, including swimming, basketball, soccer, and team handball. These findings are consistent with earlier studies by Lidor et al. (50, 51), which also found no RAE among Israeli adult elite athletes, suggesting that the effect may be less prevalent in Israeli women's sports compared to other international contexts.

International youth and professional contexts show mixed results, but they generally indicate a stronger presence of RAE than what was observed in the Israeli female basketball context. In a large-scale study of over 100,000 licensed youth players in France, a significant RAE was found across all age groups, with a clear over-representation of female players born in the first two quarters of the year (24). Similarly, in Italy, Brustio et al. (57) identified a small but statistically significant RAE among 1,535 professional female athletes across basketball, soccer and volleyball, with those born in the first quarter of the year being 1.6 times more likely to reach elite levels than those born later. In England, Kelly et al. (58) reported a noticeable RAE among girls aged 12–14 entering regional basketball talent hubs, although the effect diminished at the national level and disappeared entirely by the senior stage. Evidence of RAE was also present at high-level international youth competitions among female participants in U-16, U-18, and U-20 European Basketball Championships; however, it was less pronounced than in males and did not translate into better performance outcomes (59). Similarly, a study of Romanian junior female players found that RAE largely existed in the U-16 and U-20 age categories but not in the U-18 category, where 75% of all team members were a year younger than their teammates, yet still performed better (60). These results suggest that late-born athletes can achieve comparable performance levels when provided with equal development opportunities.

While some international contexts—particularly in youth or early development stages—show strong RAE patterns, others, especially at elite youth and senior international levels, show little or no effect (e.g., 61, 62). This variability makes the Israeli case particularly noteworthy. The findings of the current study may be partially explained by the “open door” policy commonly adopted by Israeli coaches, as proposed by Lidor et al. (52). Under this approach, not only are the most talented children selected based on their immediate performance, but also those with potential for future athletic development. Thus, sports clubs may encourage children to continue training regardless of whether they initially display the physical traits or abilities typically associated with high-level performance. These inclusive selection practices create opportunities for late bloomers—including those born late in the selection year—to participate in sports and benefit from structured training led by qualified and experienced coaches. The approach may be especially important for girls, given the significantly lower rates of female participation in sports in Israel. According to data issued by the Central Bureau of Statistics (63), only 23% of active athletes in Israel are female, with the majority (93.1%) participating in artistic gymnastics or trampolining. This results in a smaller talent pool for both competitive and recreational programs.

While the explanation offered by Lidor et al. (52) may help account for the absence of RAE among Israeli female basketball players—highlighting the local participation dynamics and coaching practices—it does not fully explain the similar lack of RAE observed among foreign players in the sample. This suggests that additional factors may be influencing talent development in women's basketball across different contexts. One such factor may be the widespread popularity of ball sports like basketball, which is highly favored among both boys and girls in Israel (64). According to CBS data (cited in 52), 48,799 children and adolescents aged 12–17 participated in various ball sports during 2017–2018, representing about 4% of that age group. Of these, 40.9% played soccer and 36.8% played basketball. Basketball enjoys similarly high levels of youth participation in countries like the U.S., as reported by Project Play (https://www.projectplay.org) and the Sports Foundation (https://www.sportsfoundation.org). In such widely played sports, early selection processes tend to be more competitive and focused on physical attributes—such as height—over relative age, as these are often perceived as better indicators of future potential. As a result, coaches may prioritize anthropometric traits during talent identification, reducing the impact of RAE even in high-demand environments.

Supporting this interpretation is the physical profile of the players in our sample. According to data from the NCD Risk Factor Collaboration (https://www.ncdrisc.org), the average height of Israeli women in 2019 was 162.22 cm (men: 175.98 cm), while in the U.S. it was 163 cm (men: 177 cm). In contrast, both Israeli and foreign female players in our study were significantly taller than these national averages, with the foreign players averaging nearly 9 cm taller than their Israeli counterparts. This height advantage likely played a critical role in their identification and progression within elite basketball programs. Taller players tend to stand out during early talent selection stages, attracting coaches' attention regardless of their relative age within the cohort. Moreover, this physical advantage continues to benefit players as they mature, improving their chances of success at both national and international levels.

### 4.2 Limitations and future research directions

The findings of this study contribute to our understanding of RAE in female basketball players. Yet a number of limitations should be addressed. First, the generalizability of these findings should be approached with caution due to the limited sample size, particularly in relation to foreign players. As noted by Lidor et al. (52), smaller sample sizes reduce the likelihood of detecting significant RAE. Yet, these authors believe the data still offer valuable insights into the theoretical and practical implications of RAE in the context of female athletes in Israel. Caution is also advised when attempting to apply these findings to other female sport settings. It should be noted that RAE is prevalent in certain female sport contexts (e.g., 24, 58), while many others have yet to be thoroughly investigated, as Cobley et al. (55) observed.

Second, this study lacked data on the players' relative age compared to their school peers, specifically whether they were older or younger than the majority of their classmates. This gap makes it challenging to draw additional conclusions about the impact of relative age on their development as athletes and on their professional careers, highlighting an area for future research.

Finally, the cross-sectional design of this study is valuable for identifying patterns related to RAE, however it limits the ability to identify the mechanisms underlying the observed patterns or understand how RAE may influence athletes' development over time. Developmental trajectories and long-term effects are essential for understanding how being relatively older or younger within an age group might impact athletes' skill progression, psychological adjustment, and career longevity. Therefore, future studies could benefit from longitudinal designs to help uncover not only if RAE persists but also how and why it affects athletes differently across their careers.

## 5 Conclusions

Contrary to trends commonly reported in both male and female sports, this study found no significant RAE among elite female basketball players. These results are consistent with prior research on Israeli female athletes and suggest that contextual factors—such as inclusive coaching approach and low female participation rates—may reduce birthdate-related selection biases. However, the absence of RAE among foreign players, despite differing sporting systems, points to the potential importance of physical attributes like height in talent identification and advancement at the elite level. In basketball, where height is a key advantage, it may outweigh the typical influence of relative age. Future studies should aim to compare these findings with data from more competitive or professionally established women's basketball leagues, where selection pressures and athlete pools may differ. Such comparisons could help clarify whether the patterns observed here reflect a broader trend or are unique to the Israeli context.

## Data Availability

The original contributions presented in the study are included in the article/Supplementary Material, further inquiries can be directed to the corresponding author.
